# The sodium–glucose cotransporter 2 inhibitor tofogliflozin prevents diabetic kidney disease progression in type 2 diabetic mice

**DOI:** 10.1002/2211-5463.13014

**Published:** 2020-11-10

**Authors:** Zi Li, Maki Murakoshi, Saki Ichikawa, Takeo Koshida, Eri Adachi, Chigure Suzuki, Seiji Ueda, Tomohito Gohda, Yusuke Suzuki

**Affiliations:** ^1^ Department of Nephrology Juntendo University Faculty of Medicine Tokyo Japan; ^2^ Department of Cellular and Molecular Neuropathology Juntendo University Graduate School of Medicine Tokyo Japan

**Keywords:** diabetic kidney disease, KK‐A*^y^* mouse, SGLT2 inhibitor, tofogliflozin

## Abstract

Trials on cardiovascular and renal outcomes in patients with type 2 diabetes have consistently demonstrated that sodium–glucose cotransporter 2 (SGLT2) inhibitors reduce the risk of diabetic kidney disease (DKD) progression. However, their renal protective mechanisms have yet to be completely understood and the effect on albuminuria reduction in animal models is controversial. We investigated these issues using KK and KK‐*A^y^* mice as a control (CTRL) and as a model for type 2 diabetes (DKD), respectively. KK‐*A^y^* mice were treated with 0.015% tofogliflozin, which is an SGLT2 inhibitor, starting at seven weeks of age for eight weeks. Compared with the CTRL mice, the DKD mice had higher HbA1c levels and albuminuria. Although tofogliflozin treatment significantly lowered HbA1c levels, it did not reverse albuminuria. Tofogliflozin treatment enhanced damage in both the glomerular (i.e., enlarged mesangial area, increased foot process effacement rate, and decreased number of WT‐1‐positive cells) and tubulointerstitial (increased protein levels of KIM‐1 and MCP‐1, increased number of macrophages, and abnormal mitochondrial morphology) areas. Our results suggest that tofogliflozin may prevent glomerular and tubulointerstitial damage, partly by ameliorating hyperglycemia, renal inflammation, and abnormal mitochondrial morphology.

## Graphical abstract

Tofogliflozin may prevent glomerular (enlarged mesangial area, foot process effacement, podocyte loss) and tubulointerstitial damage (increased expression of MCP‐1 and KIM‐1), partly by ameliorating hyperglycemia, renal inflammation, and abnormal mitochondrial morphology.

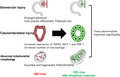

**Abbreviations**
DKD: diabetic kidney disease
EPO: erythropoietin
HbA1c: hemoglobin A1c
HIF‐1α: hypoxia‐inducible factor 1 α
KIM‐1: kidney injury molecule 1
MCP‐1: monocyte chemoattractant protein‐1
SGLT2: sodium–glucose cotransporter 2

Diabetic kidney disease (DKD) has been the most common cause of end‐stage renal disease and requires renal replacement therapy [1]. Achieving optimal glucose control and lowering of blood pressure with the use of renin–angiotensin system inhibitors can delay the progression of DKD [2]. Despite the aforementioned therapies, considerable residual risks for DKD onset and progression remain. Therefore, further measures to improve renal outcomes among patients with DKD are needed.

Approximately 90% of filtered glucose is reabsorbed in the brush border of the early proximal tubule through the sodium–glucose cotransporter 2 (SGLT2). SGLT2 inhibitors lower blood glucose level and blood pressure by inhibiting glucose and sodium reabsorption (i.e., osmotic diuresis) [3]. Given the temporary decline in estimated glomerular filtration rate shortly after SGLT2 inhibitor initiation, there were considerable concerns regarding harmful effects on the kidney early on following introduction of the medicine [4]. However, recent clinical trials on patients with type 2 diabetes have indicated that SGLT2 inhibitors have renoprotective benefits [5], which have several potential mechanisms. One hypothesis is that increased sodium concentration in the macula densa suppresses glomerular hyperfiltration through tubuloglomerular feedback response. Another is the prevention of tubulointerstitial injury by reducing excessive ATP consumption. However, such mechanisms have yet to be completely understood.

Numerous animal model studies have been performed to elucidate the effects of SGLT2 inhibitors [6, 7, 8]. However, the albuminuria‐lowering effects of SGLT2 inhibitors differed, even among similar diabetic models or therapeutic drugs. To confirm the protective effects of SGLT2 inhibitors on the kidneys, the present study investigated the renoprotective effects of the highly selective SGLT2 inhibitor tofogliflozin using KK‐*A^y^* mice as the model for type 2 diabetes.

## Methods

### Animals

The animal committee of Juntendo University Faculty of Medicine approved all animal experiments conducted herein. Five‐week‐old male KK‐*A^y^* and KK mice were purchased from CLEA Japan (Tokyo, Japan). Tofogliflozin was generously provided by Kowa Company, Ltd. (Tokyo, Japan). Starting at seven weeks of age, KK‐*A^y^* mice were administered 0.015% tofogliflozin, which was mixed in their diet for eight weeks. Fluid intake was measured every week. KK‐*A^y^* mice were then divided into the following two groups: tofogliflozin treatment (Tx group, *n* = 14) and nontreatment (DKD group, *n* = 15) groups. The nondiabetic KK mice were used as normal control (CTRL, *n* = 9). The mice were individually housed in plastic cages with free access to rodent pellet diet (CE‐2, CLEA Japan) and water throughout the experimental period. At 15 weeks of age, the mice were sacrificed for blood and tissue collection. All animals were housed under a 12‐h light/dark cycle (07:00–19:00), controlled room temperature (20–26 °C), and humidity. The mice were treated according to the guidelines for animal experimentation of Juntendo University, Tokyo, Japan (310147).

### Biochemical measurements

Body weight, food intake, water intake, and hemoglobin A1c (HbA1c) were measured at 6 and 15 weeks of age. The mice were placed in metabolic cages for 24 h for albumin/creatinine ratio measurement using DCA 2000 microalbumin–creatinine immunoassay cartridges with a DCA Vantage Analyzer (Siemens Healthcare, Erlangen, Germany). Blood HbA1c levels were analyzed using DCA 2000 HbA1c immunoassay cassette and DCA Vantage Analyzer (Siemens Healthcare). Serum tumor necrosis factor 1 (TNFR1) and TNFR2 levels were measured by an ELISA kit (R&D Systems, Minneapolis, MN, USA, MRT10, MRT20), according to the manufacturer's instructions. At least three to six blood pressure measurements were taken per session in 15‐week‐old mice using a noninvasive tail sleeve and pulse transducer system (BP‐98A; Softron, Tokyo, Japan), which were preheated for 10 min at 38 °C. A standard deviation of < 10.0 was used for blood pressure levels.

### Renal histology and immunohistochemical analysis

This study used 4% paraformaldehyde for left ventricular infusion and sagittal kidney section fixation, after which the sections were embedded in paraffin for light microscopy. Immunohistochemical studies were performed using the following commercially available antibodies: rabbit monoclonal anti‐Wilms' tumor 1 (WT1; Abcam, Cambridge, UK, ab89901); mouse monoclonal anti‐SGLT2 (Santa Cruz Biotechnology, CA, USA, sc‐393350); mouse monoclonal anti‐megalin antibody (Santa Cruz Biotechnology, sc‐515772); rabbit monoclonal anti‐TNF receptor 2 (Abcam, ab109322); and rabbit polyclonal anti‐monocyte chemoattractant protein‐1 (MCP‐1) antibody (Abcam, ab25124). For immunofluorescence, the kidneys were fixed in the same reagent that was used for light microscopy. Immunofluorescence staining with goat polyclonal anti‐kidney injury molecule 1 (KIM‐1) antibody (R&D Systems, AF1817); rat monoclonal anti‐mouse F4/80 antibody (Bio‐Rad, Hercules, CA, USA, MCA497G); rabbit monoclonal anti‐mouse cleaved caspase 3 (Cell Signaling Technology, 9661S, Beverly, MA, USA); and poststaining with 4′,6‐diamidino‐2‐phenylindole were conducted. KS400 (KS400; Carl Zeiss Vision, Munich, Germany) was used to quantitatively measure the sections. Kidney sections were stained with a periodic acid–Schiff (PAS) reagent. The PAS‐stained mesangial area on a cross‐sectional area of the glomerulus was quantitatively measured using KS400. At least 20 midsections of glomeruli were randomly selected in each group for PAS staining quantification. The number of F4/80‐positive cells, as a macrophage marker, was counted in at least 10 randomly selected fields in each mouse.

To confirm podocyte loss, the WT1‐positive cells in each group were counted in 10 randomly selected glomerular sections (*n* = 5 mice in each group). The average of the WT1‐positive cells per glomerular section was calculated.

### Transmission electron microscopy and morphometry

For electron microscopy (EM), small blocks of kidney tissue were fixed with 2% glutaraldehyde and postfixed in 1% OsO_4_ after perfusion fixation with 2% paraformaldehyde buffered with 0.1 m PB. Tissues were dehydrated in ethanol and embedded in epoxy resin. Ultrathin sections were stained with uranyl acetate and lead citrate and were examined on EM (H‐7700; Hitachi, Tokyo, Japan) at 100 kV. The rate of foot process effacement was assessed and determined by dividing the length of effacement by the length of the capillary. The mitochondrial morphology in the proximal tubules was analyzed by the long/short‐axis ratio [9]. We measured five glomeruli per mouse and five capillaries per glomerulus from five mice of each sample using imagej software (National Institute of Mental Health, Bethesda, MD, USA).

### Quantitative real‐time polymerase chain reaction

RNA was extracted from the renal cortex. Quantitative real‐time reverse transcriptase polymerase chain reaction was performed using TaqMan Gene Expression Assays (Applied Biosystems, Foster City, CA, USA) for hypoxia‐inducible factor 1 α subunit (HIF‐1α) and erythropoietin (EPO), with assay IDs Mm00468869_m1 and Mm01202755_m1, respectively. The relative mRNA level in the sample was normalized for glyceraldehyde 3‐phosphate dehydrogenase content.

### Statistical analysis

Statistical analysis was conducted using graphpad prism 7 (GraphPad Software Inc., San Diego, CA, USA). Data were expressed as mean ± standard deviation or as median (25th and 75th percentiles). Analysis of variance or unpaired *t*‐test was used to evaluate differences between means. Furthermore, Tukey's multiple comparisons test was performed for statistical comparisons, with *P* values < 0.05 considered statistically significant.

## Results

### Physiologic and biochemical parameters in each group

As shown in Table 1, the food intake level did not differ between the CTRL and DKD groups but was significantly higher in the Tx group than in the CTRL and DKD groups. Water intake level and urine volume were higher in the DKD group than in the CTRL group but were highest in the Tx group. Despite increased food intake, body weight did not differ between the Tx and DKD groups. As predicted, HbA1c levels significantly improved after tofogliflozin treatment. The serum TNFR1 levels did not differ between the CTRL and DKD groups. However, the serum TNFR2 levels were significantly higher in the DKD group than in the CTRL group and improved in the Tx group, as shown in Table 1.

**Table 1:** Effects of tofogliflozin on the physiologic and metabolic parameters in each mouse at 15 weeks of age. Data are presented as mean ± standard deviation or median (quartiles). CTRL, control group; Tx, treatment.

	CTRL group (*n* = 9)	DKD group (*n* = 15)	Tx group (*n* = 14)
Food intake (g·day^−1^)	5.7 ± 0.8	5.8 ± 0.7	8.7 ± 1.4^a^, ^b^
Water intake (g·day^−1^)	9.1 ± 2.8	13.4 ± 3.3^a^	26.1 ± 6.9^a^, ^b^
Urine volume (mL·day^−1^)	2.3 ± 1.9	6.4 ± 2.6^a^	14.0 ± 3.9^a^, ^b^
Body weight (g)	40.5 ± 2.1	47.8 ± 4.4^a^	46.7 ± 2.0^a^
Systolic blood pressure (mmHg)	109 ± 11	130 ± 12^a^	131 ± 11^a^
HbA1c (%)	5.9 ± 1.0	10.5 ± 1.3^a^	5.6 ± 0.3^b^
Urinary albumin/creatinine ratio (mg·gCr^−1^)	134 *(86,* 186)	797 (495, 938)^a^	720 (547, 1326)^a^
Serum TNFR1 (pg·mL^−1^)	847 (740, 1028)	1074 (853, 1178)	968 (787, 2239)
Serum TNFR2 (pg·mL^−1^)	5293 (4371, 5755)	6723 (5754, 8483)^a^	5349 (4513, 6183)

^a^ *P* < 0.05 vs CTRL group.

^b^ *P* < 0.05 vs DKD group

### Effects of tofogliflozin on albuminuria, mesangial expansion, and podocyte damage

**Fig. 1:**
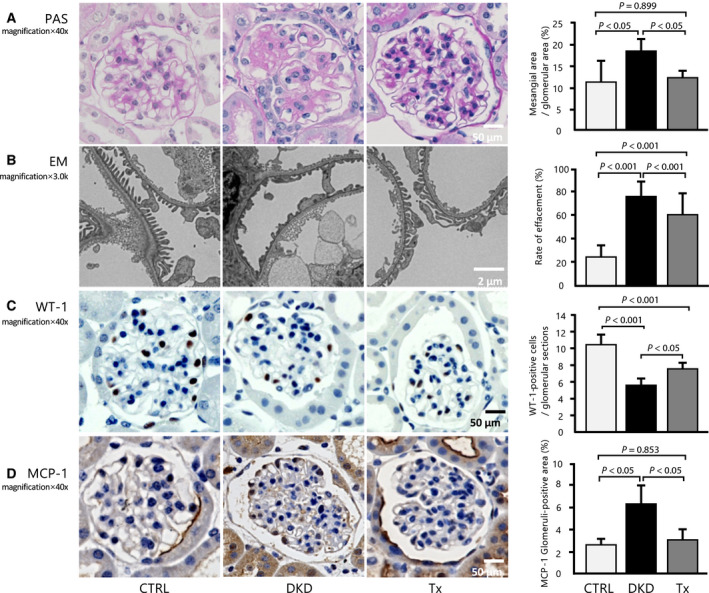
Effect of tofogliflozin on histologic improvement in the glomeruli. (A) Histologic analyses of the glomeruli after eight weeks of treatment, as shown in these representative images of the kidney mesangial matrix area (PAS) (40×, scale bar: 50 μm; *n* = 5 per group, 20 glomeruli per mouse). (B) Quantitative analysis of effacement rate (3000×, scale bar: 2 μm; *n* = 5 per group, five glomeruli and capillaries per mouse). (C) Representative micrographs show glomerular WT1 staining for each group (40×, scale bar: 50 μm; *n* = 5 per group, 20 glomeruli per mouse). (D) Inflammatory change in the glomeruli in MCP‐1 staining (40×, scale bar: 50 μm; *n* = 5 per group, at least 10 glomeruli per mouse). Data are presented as mean ± standard deviation. One‐way ANOVA was applied.

Compared with the CTRL group, the DKD group had significantly greater albuminuria and mesangial area (adjusted by glomerular area). Albuminuria level did not differ between the DKD and Tx groups, although tofogliflozin significantly ameliorated the mesangial area in the Tx group (Table 1, Fig. 1A). Tofogliflozin treatment significantly alleviated the rate of foot process effacement (Fig. 1B). The number of WT‐1‐positive cells in the Tx group was significantly larger, compared with that in the DKD group, and was almost similar to that in the CTRL group (Fig. 1C). Moreover, MCP‐1 expression in the podocytes was higher in the DKD group than in the Tx group (Fig. 1D).

### Effects of tofogliflozin on tubulointerstitial injury

**Fig. 2:**
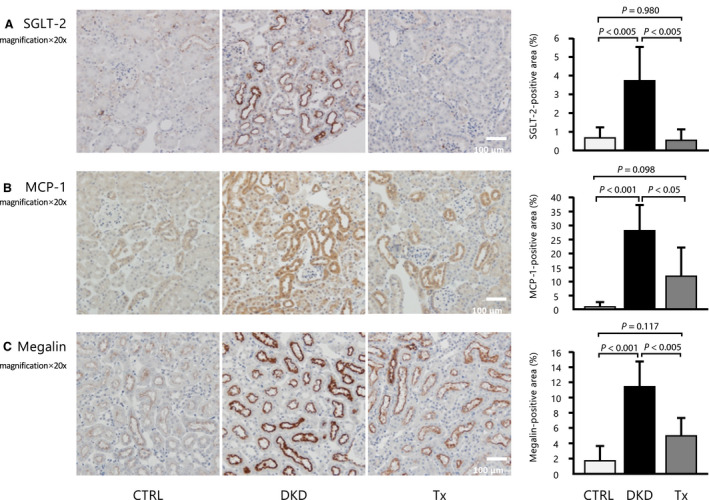
Immunohistochemistry analysis. (A) SGLT2, (B) MCP‐1, and (C) megalin staining of the renal sections from each mouse (20×, scale bar: 100 μm; *n* = 5 per group, at least 10 randomly selected renal sections per mouse). Data are presented as mean ± standard deviation. One‐way ANOVA was applied.

SGLT2 expression at the proximal tubular epithelia of segments S1 and S2 was significantly upregulated in DKD group and was almost ameliorated after tofogliflozin treatment (Fig. 2A).

**Fig. 3:**
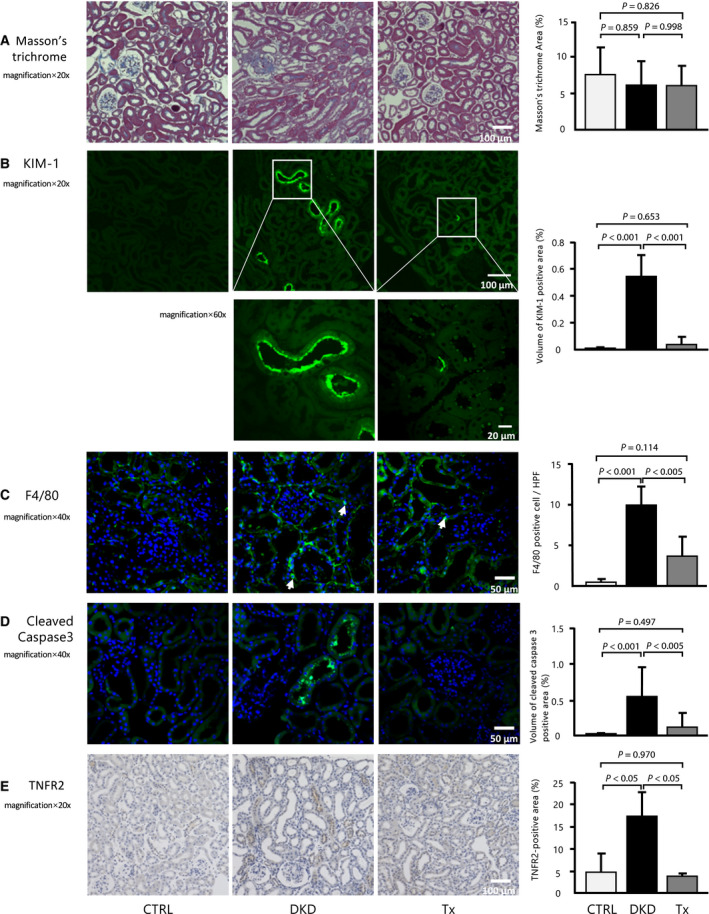
Effect of tofogliflozin administration on tubulointerstitial injury. (A) Immunohistochemistry analysis on (A) Masson's trichrome staining (20×, scale bar: 100 μm). Immunofluorescence analyses of the renal sections of each mouse with (B) KIM‐1 (green) and DAPI (blue) (20×, 60×, scale bar: 100, 20 μm). (C) F4/80 (40×, scale bar: 50 μm), (D) cleaved caspase 3 (40×, scale bar: 50 μm), and (E) TNFR2 (20×, scale bar: 100 μm) (*n* = 5 per group, at least 10 randomly selected renal sections per mouse). Data are presented as mean ± standard deviation. One‐way ANOVA was applied.

Immunohistochemical staining showed that compared with the CTRL group, the DKD group had higher tubulointerstitial protein levels of MCP‐1, which were partly ameliorated by tofogliflozin treatment (Fig. 2B). Megalin, which plays a critical role in albumin reabsorption, showed an expression pattern that was similar to that of SGLT2 (Fig. 2C). This could explain the unaltered albuminuria despite improvements in glomerular and tubulointerstitial damage. Masson's trichrome staining showed that interstitial fibrosis was not significantly different among the three groups (Fig. 3A). Immunofluorescence revealed that the KIM‐1 protein, which is a tubulointerstitial injury marker, was hardly observed in the CTRL and Tx groups but was found in some proximal tubules of the DKD group (Fig. 3B). Tubulointerstitial inflammation, which is characterized by infiltration of macrophages, is an important step in the progression of DKD, because it is closely associated with renal fibrosis. F4/80‐positive cells were mainly localized in the peritubular capillaries and tubulointerstitium. The number of F4/80‐positive cells was significantly increased in the tubulointerstitium of the DKD group and significantly improved after tofogliflozin treatment (Fig. 3C). Detection of cleaved caspase‐3‐positive staining of the proximal tubules was very faint in the CTRL group and was significantly increased in the DKD group. Importantly, the increased cleaved caspase‐3 expression was significantly reduced by tofogliflozin treatment (Fig. 3D). Because the serum levels of TNFR2 tended to improve with tofogliflozin, we stained the kidney for TNFR2 expression. Similar to the serum level, kidney TNFR2 protein expression was enhanced in the DKD group and was improved by tofogliflozin (Fig. 3E).

**Fig. 4:**
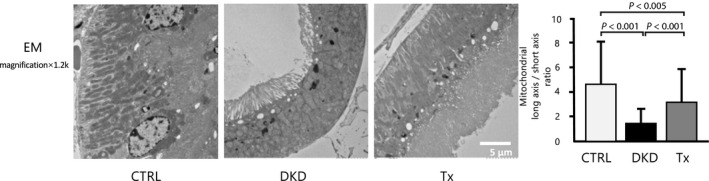
Electron microscopy analysis of mitochondrial morphology. On the long/short‐axis ratio, Tx mice were significantly relieved compared with DKD mice in proximal tubule (1200×, scale bar: 5 μm; *n* = 3 per group, at least 10 randomly selected fields per mouse). Data are presented as mean ± standard deviation. One‐way ANOVA was applied.

Mitochondrial morphology on transmission EM was compared between the DKD and CTRL groups (Fig. 4). Mitochondrial swelling was ameliorated by tofogliflozin treatment.

**Fig. 5:**
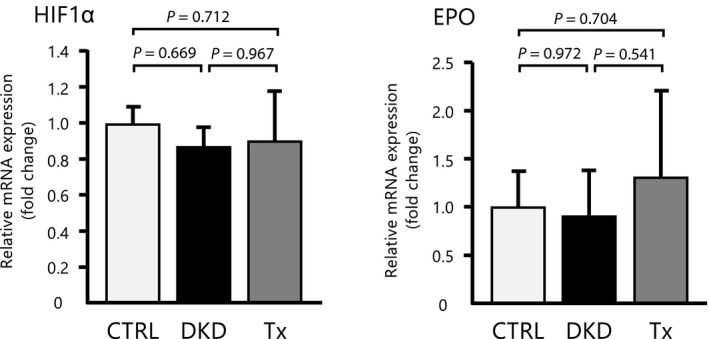
Real‐time polymerase chain reaction analysis of EPO and the hypoxia‐inducible factor 1 alpha subunit mRNA in the kidneys. mRNA levels of HIF‐1α and EPO did not differ between DKD and Tx mice (*n* = 5 per group). Data are presented as mean ± standard deviation. One‐way ANOVA was applied.‬

We also hypothesized that tofogliflozin could improve hypoxia‐induced tubulointerstitial damage and eventually lead to anemia by preventing glucose reabsorption from the proximal tubule. However, the mRNA levels of EPO and HIF‐1α in the kidney did not differ among the three groups (Fig. 5).

## Discussion

In this study, we provided experimental evidence that SGLT2 inhibition with tofogliflozin promoted renoprotective effects in a mice model of type 2 diabetes. Despite the absence of decrease in albuminuria, improved abnormal glomerular phenotypes, such as glomerular hypertrophy and podocyte loss, and tubulointerstitial inflammation were observed after tofogliflozin administration.

SGLT2 inhibitors cause direct weight loss through glucose excretion in the kidney. Contrary to most research, this present study showed that the body weight of the Tx mice did not improve, probably because of their increased food intake levels. Similarly, Tanaka *et al*. [10] reported that food intake was approximately 25% greater in the SGLT2 treatment group than in the vehicle group and that weight loss was not observed after SGLT2 inhibitor treatment. If we performed the method using pair feeding, the body weight was likely to have decreased. In the present study, although weight loss was not observed, glomerular injury improved with tofogliflozin treatment. Immunohistochemical analysis revealed that DKD mice had higher SGLT2 protein expression in the brush border of the proximal tubules, compared with that in the Tx mice, although similar staining patterns were observed in both groups. Studies showed that a hyperglycemic state upregulated SGLT2 expression, which resulted in further increase in glucose and sodium reabsorption [11]. Excess glucose entry into tubular epithelial cells through SGLT leads to increased expression of inflammatory molecules, such as IL‐6 and MCP‐1, and macrophage infiltration [12, 13]. Therefore, enhanced SGLT2 expression under hyperglycemic conditions promotes persistent inflammation. Accordingly, our results showed that tofogliflozin improved the expressions of SGLT2, MCP‐1, and KIM‐1 in the kidney and the number of F4/80‐positive cells, thereby demonstrating that tofogliflozin can suppress tubular inflammation and prevent exacerbation of tubulointerstitial damage. In this study, the mechanism of reduced SGLT2 protein expression with SGLT2 inhibitor administration was not clear. Umino *et al*. [14] reported that renal SGLT2 expression increased in diabetic db/db mice, compared with that in nondiabetic db/m mice, and this increase was attenuated by SGLT2 inhibitor treatment in db/db mice. They examined SGLT2 expression in the tubule cells using a two‐chamber culture system. SGLT2 expression after the addition of high glucose increased on the basolateral side but not on the apical side. Other studies on cultured human proximal tubule cells reported enhanced SGLT2 expression with insulin stimulation but not with high‐glucose stimulation [15]. Hyperinsulinemia in DKD mice may contribute to increased SGLT2 expression, which may be reduced by SGLT2 treatment. Further research is needed to confirm this finding.

Considering that the lack of change in albuminuria despite improved glomerular hypertrophy and podocyte loss, the present study focused on megalin, which is a molecule involved in protein reabsorption. Accordingly, immunohistochemical staining showed that megalin level was higher in DKD mice than in CTRL mice and was ameliorated by tofogliflozin treatment. Tofogliflozin may decrease albumin uptake in the proximal tubules by reducing megalin expression, resulting in albuminuria. However, reports on renal megalin expression in diabetes have been conflicting [16, 17, 18]. Takiyama *et al*. [19] reported that luseogliflozin, which is another SGLT2 inhibitor, ameliorated hyperglycemia but not albuminuria in diabetic db/db mice. Moreover, they showed that luseogliflozin inhibited albumin uptake by decreasing megalin expression. According to Bryniarski *et al*. [20], insulin treatment under high‐glucose conditions significantly increased the protein and mRNA expressions of megalin, as well as albumin endocytosis in the proximal tubule cell line. This result suggested that insulin, similar to SGLT2 inhibitor, might be involved in the regulation of megalin expression. Considering that KK‐*A^y^* spontaneously showed the association between type 2 diabetes and hyperinsulinemia, the increased expression of megalin might have been related to insulin levels. Given that the megalin‐mediated endocytic handling of substances filtered by the glomeruli is involved in tubular injury [21], tofogliflozin might have exerted its renoprotective effects by inhibiting the protein expression of megalin.

Recent reports showed that SGLT2 inhibitors could normalize glucose handling and energy consumption in the proximal tubule [22]. The proximal tubules in the kidney contain many mitochondria. Mitochondrial dysfunction leads to decreased ATP production and causes podocyte injury, tubular epithelial cell damage, and endothelial dysfunction. We found that a diabetic condition damaged the proximal kidney tubule and showed morphologic evidence of mitochondrial damage and that tofogliflozin partly reversed these damages to a normal status. SGLT2 inhibitors prevented mitochondrial dysfunction, suggesting that they might improve energy supply.

Increased sodium reabsorption by the proximal tubules decreases sodium delivery to the macula densa, which induces glomerular hyperfiltration through the tubule‐glomerular feedback mechanism [23]. Given the high‐energy requirement for reabsorption of electrolytes and organic solutes, the proximal tubules consume the most oxygen in the kidneys [24] and are, therefore, particularly susceptible to hypoxia. Glomerular hyperfiltration results in increased oxygen demand within the tubular cells. SGLT2 inhibition remedies this energy imbalance by reducing the workload of proximal tubular cells. However, the present study found no differences in hypoxia‐related mRNA expression among the three groups analyzed. In this model, we presumed that inflammation had a greater influence on the mitigation of histologic aggravation in the kidneys, compared with that of hypoxia.

Circulating TNFR level is known to be a strong predictive biomarker of kidney function [25]. We previously reported that serum TNFR2 level but not serum TNFR1 level was reduced by treatment in patients with IgA nephropathy [26]. Heerspink *et al*. [27] showed that SGLT2 inhibitors attenuated the plasma levels of TNFR1. Likewise, in the present study, we showed that serum TNFR2 level decreased after SGLT2 inhibitor treatment. These implied a renoprotective effect of tofogliflozin.

## Conclusions

Our results suggested the renoprotective effects of tofogliflozin against glomerular and tubulointerstitium damage in diabetic mice. These effects were at least partly related to hyperglycemia, mitochondrial dysfunction, and inflammation. Further studies are needed to confirm these findings.

## Author contributions

ZL acquired and analyzed the data. MM and TG designed the project, interpreted the data, and wrote the manuscript. SI, TK, EA, and CS acquired the data. YS reviewed the manuscript.

## Conflicts of interest

The authors declare no conflict of interest.

## Data Availability

All data generated or analyzed during this study are included in this article. Data will be available from the corresponding author upon reasonable request.
